# Associations between ambient particulate matter exposure and the prevalence of arthritis: Findings from the China Health and Retirement Longitudinal Study

**DOI:** 10.1371/journal.pone.0327695

**Published:** 2025-07-08

**Authors:** Yuntian Ye, Kuizhi Ma, Aifeng Liu

**Affiliations:** 1 Orthopedics Department, First Teaching Hospital of Tianjin University of Traditional Chinese Medicine, Tianjin, China; 2 National Clinical Research Center for Traditional Chinese Medicine and Acupuncture, Tianjin, China; National Autonomous University of Mexico Institute of Geophysics: Universidad Nacional Autonoma de Mexico Instituto de Geofisica, MEXICO

## Abstract

**Background:**

Arthritis is a leading cause of global disability, but its etiology is complex and has not yet been fully understood. Recent studies suggest that air pollution is potentially linked to arthritis onset, although current research predominantly focuses on the effects of particulate matter (PM_2.5_). There is a lack of comprehensive analysis regarding the influence of various particulate pollutants and their key constituents on arthritis prevalence. This research used China Health and Retirement Longitudinal Study (CHARLS) data and explored the associations between arthritis prevalence and different particulate matters (PM_2.5_, PM_10_, and PM_1_, ≤ 2.5 micrometers, ≤ 10 micrometers, and ≤ 1 micrometer in diameter, respectively), as well as ammonium (NH_4_) and nitrate (NO_3_), with gaseous pollutants [such as sulfate (SO_4_) and ozone (O_3_)] as a reference.

**Methods:**

This study was conducted based on the 2015 CHARLS cross-sectional data, and 3,802 participants were included. The levels of air pollution exposure were estimated using a spatial-temporal extreme random forest model, integrating ground monitoring, remote sensing data, and model simulations, encompassing PM_1_, PM_2.5,_ PM_10_, NH_4_, NO_3_, O_3_, and SO_4_ concentrations. The association of air pollution with arthritis prevalence was assessed utilizing generalized linear models (GLM), while adjusting for various confounding variables.

**Results:**

Long-term exposure to PM₁, PM₂.₅, PM₁₀, NH₄ ⁺ , and NO₃ ⁻ was positively associated with self-reported arthritis prevalence. Specifically, each interquartile range (IQR) increase in PM₁ corresponded to 4.4% higher odds of arthritis (odds ratio [OR] per IQR: 1.044; 95% confidence interval [CI]: 1.011–1.070), indicating a modest association. Subsequent ORs per IQR for PM₂.₅ and PM₁₀ were 1.019 (95% CI: 1.004–1.036) and 1.012 (95% CI: 1.002–1.021), respectively, reflecting similar but smaller positive associations, whereas NH₄⁺ and NO₃ ⁻ showed moderate associations with ORs per IQR of 1.143 (95% CI: 1.017–1.285) and 1.082 (95% CI: 1.011–1.159), respectively. These findings were robust to sensitivity analyses.

**Conclusion:**

To our knowledge, this is the first study to identify a significant association between long-term exposure to PM₁, PM₂.₅, PM₁₀ and the secondary inorganic aerosol constituents NH₄ and NO₃, and the prevalence of self-reported arthritis in middle-aged and older adults. However, owing to its cross-sectional design, the absence of subtype differentiation and reliance on self-reported diagnoses, these findings may be influenced by reverse causation and measurement error, and should therefore be interpreted with caution.

## Introduction

Arthritis is a comprehensive category of ailments, encompassing over a hundred distinct rheumatic diseases and associated conditions. As a major cause of global disability, arthritis profoundly affect patient’s life quality, as well as the broader socio-economic landscape [1,2]. Over the past three decades, arthritis global prevalence and incidence of arthritis have gained an increase of 7.4% and 8.2%, respectively [3]. Arthritis primarily encompasses osteoarthritis (OA) and rheumatoid arthritis (RA). Although OA and RA share similar clinical presentations, their prevalence and treatment approaches differ markedly. OA has traditionally been viewed as a “wear-and-tear” disorder chiefly affecting cartilage, but recent research has shown that it is a complex inflammatory disease involving interactions among cartilage, bone, and synovium [4]. RA, by contrast, is an autoimmune disorder characterized by chronic synovial inflammation that leads to joint destruction and loss of function [5]. In OA, synovial fibroblasts play a key role in maintaining joint homeostasis, yet the mechanisms underlying their interactions across distinct cellular subtypes and the initiation of specific signaling pathways remain incompletely understood [6]. The pathophysiology of RA involves a complex imbalance in immune regulation; studies indicate that interactions between synovial fibroblasts and other immune cells are important in disease progression, although the primary pathogenic mechanisms have yet to be fully elucidated [7]. With the aging population, shifts in lifestyle, and advancements in disease diagnosis, the global prevalence of arthritis is projected to continue its steady rise. However, arthritis often arises from a confluence of various factors, and its pathogenic mechanisms remain incompletely understood. Studies have shown that factors such as obesity and metabolic syndrome are closely associated with arthritis prevalence. Obesity not only elevates the risk of arthritis but is also linked to greater disease activity and reduced quality of life [8]. Moreover, components of metabolic syndrome—such as body mass index and waist circumference—demonstrate a positive correlation with arthritis prevalence [9]. Environmental factors, including air pollution, dietary habits, and infections, as well as hormonal and reproductive influences, may also contribute to the development of arthritis [10]. Consequently, arthritis prevalence is determined by the interplay of multiple determinants, and elucidating these underlying factors is essential for designing effective intervention strategies to reduce prevalence and alleviate its burden on individuals and society.

Air pollution stands as one of the most pressing public health challenges globally. Over the past decade, China has been frequently and persistently confronted with widespread smog events [11,12], with more than 1.3 billion people nationwide facing health risks due to exposure to fine particulate matter that exceeds safety standards [13]. Previous studies have confirmed that air pollution may trigger diverse health issues, including asthma [14], cardiovascular diseases [15], type 2 diabetes [16], and arthritis [17]. Notably in the context of arthritis, accumulating evidence suggests that air pollution may constitute a critical environmental determinant influencing both disease onset and progression. A study disclosed that individual and combined PM_2.5_ components exert detrimental effects on rheumatoid arthritis, with OM, NO_3_, and NH_4_ exposure demonstrating U-shaped or J-shaped exposure-response relationships in modulating disease development [18]. Furthermore, a population-based investigation established a significantly heightened association between PM_2.5_ exposure and arthritis risk among individuals with type 2 diabetes mellitus compared to their non-diabetic counterparts [16]. Of particular significance, a nationwide case-crossover study in China substantiated those incremental concentrations of PM_2.5_, PM_10_, NO_2_, SO_2_, O_3_, and CO exhibited significant correlations with elevated osteoarthritis-related hospitalization rates [19]. Collectively, these findings underscore that chronic exposure to air pollutants, particularly PM_2.5_, may potentiate the risk profile for arthritis pathogenesis. Nevertheless, existing research has predominantly focused on the impact of PM_2.5_ particles on arthritis incidence, whereas the association between comprehensive particulate pollutants and arthritis prevalence rates remains unexplored. Therefore, our research aimed to utilize the China Health and Retirement Longitudinal Study (CHARLS) to investigate the association of arthritis prevalence with long-term exposure to particulate pollutants (PM_1_, PM_2.5_, and PM_10_) and key components of PM_2.5_ [ammonium (NH_4_) and nitrate (NO_3_)], simultaneously incorporating certain gaseous pollutants [sulfate (SO_4_) and ozone (O_3_)] as references.

## Methods

### Research subject

CHARLS is a nationwide cohort study of Chinese adults (≥45 years) and their spouses, designed to inform public health policy. Employing multi-stage, stratified, probability-proportional-to-size sampling across 150 counties in 28 provinces, the study enrolled a total of 17,708 participants.

**Fig 1:**
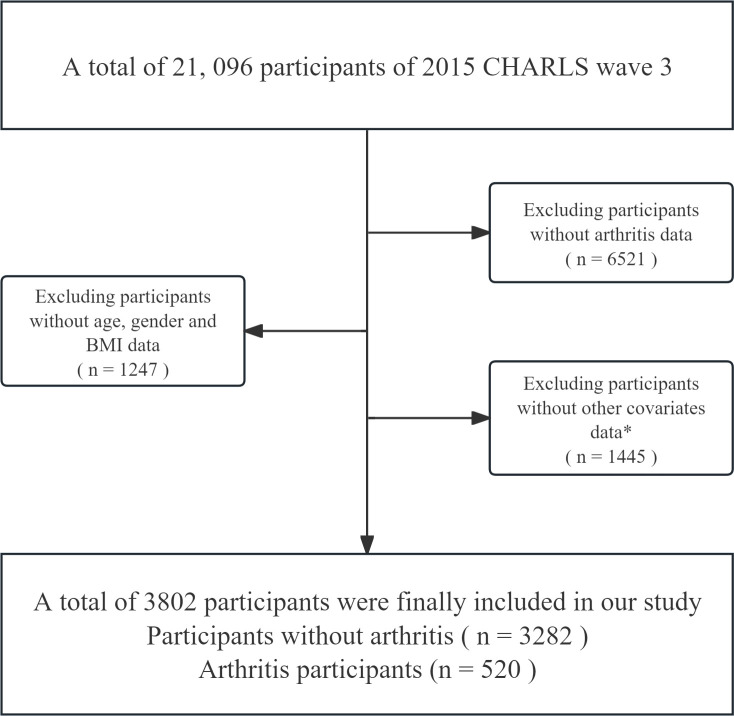
Flow chart. Flow chart of the selection of study participants. Excluded participants without arthritis data (n = 6521), without age/gender/BMI data (n = 1247), and without other covariates data (n = 1445) *; final sample = 3802 (participants without arthritis n = 3282; arthritis participants n = 520). *Other covariates: residential area, education level, marital status, annual household expenditure, smoking status, alcohol consumption, cooking fuel type, diabetes, and hypertension.

The baseline survey (2011–2012) was followed by biennial CAPI interviews covering health, socioeconomic, lifestyle factors and life-course exposures. Because China’s air-pollution data begin in 2013, we analyzed the 2015 cross-sectional wave, yielding 3 802 eligible participants (520 with arthritis; 3282 controls; Fig 1). Ethical approval for the original CHARLS project was granted by the Peking University Biomedical Ethics Committee (IRB00001052–11015), and all participants provided written informed consent. Our current work is a secondary analysis of the fully anonymized, publicly available CHARLS data.

### Air pollution exposure evaluation

Artificial intelligence was employed to assess the comprehensive ground-level concentrations of PM_1_, PM_2.5_, PM_10_, NO_3_, NH_4_, O_3_, and SO_4_ at a spatial grid resolution of 0.1 micrometers (10 km) from 2013 to 2015. These data were sourced through the China High-Resolution Air Pollution Database (CHAP) dataset (https://weijing-rs.github.io/product.html). In brief, the assessment evaluated these daily concentrations of air pollutants through integrating ground monitoring data, remote sensing products, atmospheric reanalysis data, and model simulations, as well as employing a spatiotemporal extreme random forest (STET) model. In the primary effect analysis, annual air pollution exposure calculation was conducted based on each county-level residence of survey subjects, with a 3-year average concentration prior to CHARLS wave 3 for long-term exposure, while a 2-year average concentration was utilized for sensitivity analysis [20].

**Fig 2:**
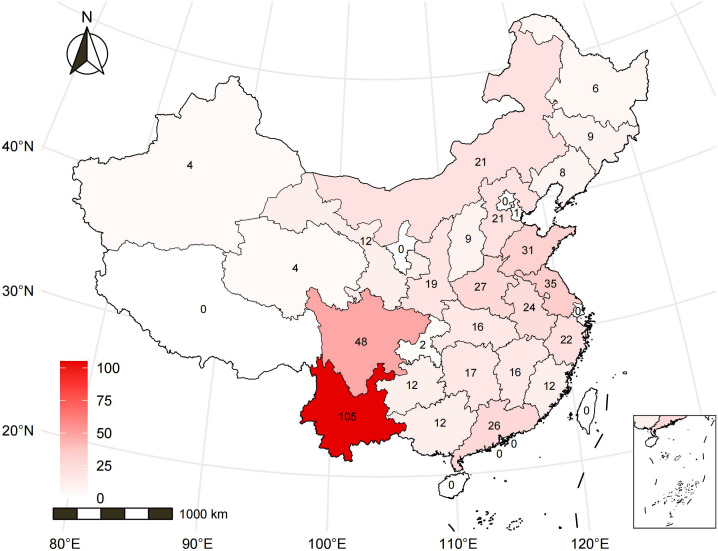
The geographical distribution of 520 middle-aged and older adults in 27 provinces of China. Distribution of arthritis prevalence in 2015, where the darker the color, the higher the number of people with the disease.

The geographic data underlying the maps in Figs 1 and 2 were obtained from the open-source geographic data service provided by AntV L7, developed by Ant Group (https://l7.antv.antgroup.com/custom/tools/map). These data are sourced from publicly available shapefiles.

### Outcome measurements

Determination of arthritis diagnosis was conducted according to self-reports from the baseline and follow-up surveys (“Have you ever been diagnosed with arthritis or rheumatism by a doctor?”). If participants answered “Yes” to this question, they were classified as having arthritis [21].

### Definition of covariates

This analysis controlled for a range of potential confounders, including sociodemographic and socioeconomic indicators, as well as participants’ health behaviors, lifestyle characteristics, and clinical comorbidities.

Based on established arthritis risk factors and potential confounders, we included the following covariates: Sociodemographic data consisted of age and gender. Socioeconomic data encompassed residence (rural; urban), education level (“primary school or below”; “secondary school and above”), and marital status (“married and living with spouse”; “married but not living with spouse”; “single, divorced, or widowed”). Health behaviors and lifestyle variables were smoking (“non-smoker”; “smoker”), alcohol (“non-drinker”; “drinks less than once a month”; “drinks more than once a month”), and indoor air pollution, which could be differentiated by cooking fuels. Cooking fuels were categorized into solid (wood, coal, and crop residues) and clean fuels (natural gas, biogas, and liquefied petroleum gas) [22]. Diabetes was assessed using the following criteria: use of diabetic medication, diabetes history, fasting plasma sugar level ≥ 126 mg, and HbA1c ≥ 6.5% [23]. Hypertension was assessed by the following indicators: systolic and diastolic blood pressure ≥ 140 mmHg and 90 mmHg, respectively, hypertension history, and antihypertensive medication.

Among these, age and sex are established risk factors for arthritis [24–26]; smoking history is a recognized risk factor for rheumatoid arthritis [27], although its broader impact on arthritis remains debated; and diabetes has been linked to increased arthritis risk [28]. These variables were included as covariates to control for their potential confounding effects on the primary analyses.

### Statistical analyses

Continuous and categorical data were described in the form of mean ± standard deviation (SD) and count data (percentage), separately. The associations between air pollutants were examined utilizing Pearson analysis.

Influence of air pollution on arthritis prevalence was investigated using a generalized linear model (GLM). The effect estimates and their 95% confidence intervals (CI) were expressed as odds ratios (OR) for arthritis corresponding to an increase in air pollutants by each interquartile range (IQR). In adjusted model 2, meteorological factors, sociodemographic (age and sex) and socioeconomic variables (residence, education level, marital status, and annual household expenditure) were accounted for. Finally, in adjusted model 3, health behaviors and lifestyle factors (smoking status, alcohol consumption, diabetes, hypertension, and cooking fuel use) were also adjusted for. Additionally, standardized concentrations of air pollution were incorporated to the model; different air pollutants’ OR was compared for determining the component of air pollution with stronger association with elevated arthritis prevalence. McFadden R² was used to assess the goodness-of-fit of the models. It is a measure similar to R² in linear regression and compares the log-likelihood of the fitted model to that of a baseline model with no predictors. Higher values indicate better model fit.

Furthermore, our results’ robustness was validated through sensitivity analyses. Firstly, the GLM model was rerun using average air pollutant concentrations from 2013 to 2014 for analysis. Secondly, a regional classification variable (“East”; “Central”; and “West”) was adjusted for to account for geographic differences in economic development. Additionally, survey subjects whose their residence changed (2011–2015) were excluded. Finally, the association of air pollution with arthritis was examined using a log-binomial Poisson regression analysis.

Statistical analyses were conducted in R version 4.4.2. Generalized linear models were fitted using the glm() function in the stats package, and McFadden’s pseudo-R² was obtained via the pR2() function in the pscl package.

## Results

### Characteristics of participants

**Fig 3:**
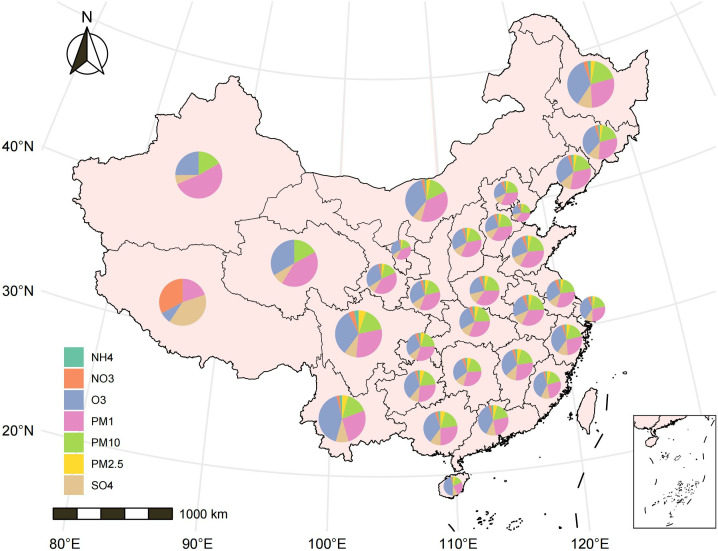
Changes in average air pollution concentrations in participants’ cities, 2013–2015. Map of differences in air pollution concentrations in the cities where participants lived from 2013 to 2015. A larger area occupying a larger proportion of the circle means a greater increase in air pollution concentration.

This research enrolled 3,802 individuals (mean age 59.1 ± 10.7 years) from CHARLS from prefecture-level cities across 27 Chinese provinces (Figs 2, 3, Table 1). In the arthritis group, urban residents had a remarkably higher proportion relative to rural residents (48.1% vs. 32.9%, *p* < 0.001). Additionally, with regard to cooking fuel use, arthritis participants using clean fuels was dramatically more than those using solid fuels (74.5% vs. 50.7%, *p* < 0.001).

**Table 1:** Basic characteristics of participants.

Characteristics	Participants without arthritis (n = 3282)	Arthritis (n = 520)	*P-*value
**Age**	62.2 (9.7)	56.4 (10.8)	< 0.001
**Sex**			< 0.001
Male	1403 (42.7)	287 (55.2)	
Female	1879 (57.3)	233 (44.8)	
**BMI, kg/m** ^**2**^	24.6 (16.3)	23.9 (3.5)	0.358
**Residence**			< 0.001
Rural	2202 (67.1)	270 (51.9)	
Urban	1080 (32.9)	250 (48.1)	
**Marital status**			< 0.001
Married and living with a spouse	2555 (77.9)	449 (86.5)	
Married but living without a spouse	188 (5.7)	24 (4.6)	
**Education Status**			< 0.001
Elementary school or below	2471 (75.3)	331 (63.7)	
Middle school or above	811 (24.7)	189 (36.3)	
**Smoking Status**			0.066
Non-smoker	1911 (58.2)	280 (53.8)	
Smoker	1370 (41.8)	240 (46.2)	
**Drinking Status**			< 0.001
Drink but less than once a month	279 (8.5)	46 (8.9)	
Drink more than once a month	762 (23.3)	167 (32.2)	
Non-drinker	2228 (68.2)	305 (58.9)	
**Hypertension**			< 0.001
Yes	865 (49.3)	176 (62.2)	
No	890 (50.7)	107 (37.8)	
**Diabetes**			< 0.001
Yes	1728 (76.5)	274 (86.4)	
No	531 (23.5)	43 (13.6)	
**Cooking Fuel Use**			< 0.001
Clean fuel	984 (50.7)	205 (74.5)	
Solid fuel	958 (49.3)	70 (25.5)	
**Annual Household** **Expenditure**	12579.8 (19418.9)	16204.5 (24891.6)	0.002
**Regional Categories**			< 0.001
East	844 (25.7)	184 (35.5)	
Midland	1150 (35.0)	171 (32.9)	
West	1288 (39.2)	164 (31.6)	

Abbreviations: BMI, body mass index.

According to the average concentrations of 7 air pollutants (Table 2, Figs 2, 3), the 3-year average exposures to environmental NH_4_, NO_3_, O_3_, PM_1_, PM_10_, PM_2.5_, and SO_4_ were 6.67 ± 2.07 (μg/m³), 9.02 ± 3.60 (μg/m³), 83.62 ± 7.10 (μg/m³), 27.71 ± 8.37 (μg/m³), 91.12 ± 30.79 (μg/m³), 50.88 ± 15.04 (μg/m³), and 10.89 ± 2.67 (μg/m³), respectively. The annual exposure levels of PM₂.₅ and PM₁₀ notably exceeded the World Health Organization’s Air Quality Guidelines (AQG 2021; PM₂.₅: 5 μg/m³, PM₁₀: 10 μg/m³) and the secondary limits of China’s national standard Environmental Air Quality Standard (GB 3095−2012; PM₂.₅: 35 μg/m³, PM₁₀: 70 μg/m³).

**Table 2:** Descriptive statistics 3-years average levels of air pollution.

Air pollution	Mean	SD	P25	P50	P75	IQR
NH_4_ (μg/m³)	6.67	0.53	6.45	6.82	6.95	0.50
NO_3_ (μg/m³)	9.02	0.92	8.66	9.32	9.53	0.87
O_3_ (μg/m³)	83.62	2.27	82.51	83.89	84.65	2.14
PM_1_(μg/m³)	27.71	2.65	26.37	28.30	28.88	2.51
PM_10_(μg/m³)	91.12	8.83	87.75	93.88	96.21	8.46
PM_2.5_(μg/m³)	50.88	4.94	48.58	52.08	53.28	4.70
SO_4_ (μg/m³)	10.89	0.95	10.52	11.21	11.41	0.89

Abbreviations: SD, standard deviation; P25, P50, P75, Lower, median and upper quartiles of variables; IQR, inter-quartile range; NH₄, Ammonium; NO₃, Nitrate; O₃, Ozone; PM_1_, Particulate Matter 1; PM_10_, Particulate Matter 10; PM_2.5_, Particulate Matter 2.5; SO₄, Sulfate.

### Association of air pollution with arthritis prevalence

As shown in Table 3, we analyzed the statistical association between different air pollutants and arthritis prevalence. Logistic regression analyses demonstrated significant associations between long-term air pollution exposure and arthritis prevalence across sequential adjustment models. In crude models, particulate matter (PM₁: OR=1.019, 95% CI:1.008–1.030, *P* < 0.001; PM₁₀: OR=1.005, 95% CI:1.002–1.009, *P* < 0.001; PM₂.₅: OR=1.008, 95% CI:1.002–1.014, *P* = 0.005) exhibited robust associations, while secondary inorganic aerosols (NH₄: OR=1.024, 95% CI:0.973–1.075, *P* = 0.322; NO₃: OR=1.017, 95% CI:0.989–1.046, *P* = 0.218) showed non-significant trends. After adjusting for demographic, socioeconomic, lifestyle, and health-related covariates, the fully adjusted model revealed that each IQR increase in exposure was associated with elevated arthritis odds: 14.3% for NH₄ (OR=1.143, 95% CI:1.017–1.285, *P* = 0.024), 8.2% for NO₃ (OR=1.082, 95% CI:1.011–1.159, *P* = 0.022), 4.4% for PM₁ (OR=1.044, 95% CI:1.011–1.070, *P* = 0.005), 1.9% for PM₂.₅ (OR=1.019, 95% CI:1.004–1.036, *P* = 0.013), and 1.2% for PM₁₀ (OR=1.012, 95% CI:1.002–1.021, *P* = 0.014). NH₄ and NO₃ associations emerged only after full adjustment, suggesting confounding by socioeconomic or behavioral factors in earlier models. Furthermore, the McFadden R² values for the associations between PM_2.5_, PM_10_, PM_1_, NO_3_, and NH_4_ with arthritis were 0.085, 0.085, 0.088, 0.083, and 0.083, respectively (Table 4). McFadden R² is a goodness-of-fit measure for categorical models.

**Table 3:** Associations between air pollution and arthritis.

Air Pollution	OR and 95%CI	*P* – value
**O**_**3**_ (IQR:2.14 μg/m³)		
Crude Model	1.042(1.026, 1.058)	6.454
Adjusted Model 1	1.039(1.020, 1.059)	5.183
Adjusted Model 2	1.050(1.025, 1.114)	8.326
**SO**_**4**_ (IQR:0.89 μg/m³)		
Crude Model	0.999(0.962, 1.037)	0.964
Adjusted Model 1	1.018(0.974, 1.064)	0.425
Adjusted Model 2	1.063(0.978, 1.156)	0.147
**NH**_**4**_ (IQR:0.5 μg/m³)		
Crude Model	1.024(0.973,1.075)	0.322
Adjusted Model 1	1.045(0.985,1.108)	0.138
Adjusted Model 2	1.143(1.017,1.285)	0.024
**NO**_**3**_ (IQR:0.87 μg/m³)		
Crude Model	1.017(0.989,1046)	0.218
Adjusted Model 1	1.023(0.989,1.059)	0.175
Adjusted Model 2	1.082(1.011,1.159)	0.022
**PM**_**1**_ (IQR:2.51 μg/m³)		
Crude Model	1.019(1.008,1.03)	< 0.001
Adjusted Model 1	1.022(1.009,1.036)	< 0.001
Adjusted Model 2	1.044(1.011,1.07)	0.005
**PM**_**10**_ (IQR:8.46 μg/m³)		
Crude Model	1.005(1.002,1.009)	< 0.001
Adjusted Model 1	1.006(1.001,1.01)	0.005
Adjusted Model 2	1.012(1.002,1.021)	0.014
**PM**_**2.5**_ (IQR:4.70 μg/m³)		
Crude Model	1.008(1.002,1.014)	0.005
Adjusted Model 1	1.009(1.002,1.017)	0.009
Adjusted Model 2	1.019(1.004,1.036)	0.013

Notes: Model 1, crude model, without adjustment; Model 2, adjusted for age, sex, residence, education level, marital status, and annual household expenditure; Model 3, adjusted for age, sex, residence, education level, marital status, annual household expenditure, smoking status, alcohol consumption, diabetes, hypertension, and cooking fuel use.

Abbreviations: IQR, inter-quartile range; OR, odds ratio; 95% CI, 95% Confidence Interval.

**Table 4:** Deviation analysis of the association between air pollution and arthritis.

Model	Log-Likelihood	Log-Likelihood of the Null Model	McFadden R²
NH_4_	−241.161	−263.177	0.083
NO_3_	−241.108	−263.177	0.083
O_3_	−240.227	−263.177	0.087
PM_1_	−239.891	−263.177	0.088
PM_10_	−240.743	−263.177	0.085
PM_2.5_	−240.689	−263.177	0.085
SO_4_	−242.678	−263.177	0.077

Abbreviations: NH₄, Ammonium; NO₃, Nitrate; O₃, Ozone; PM_1_, Particulate Matter 1; PM_10_, Particulate Matter 10; PM_2.5_, Particulate Matter 2.5; SO₄, Sulfate.

Notes: LL = Log-likelihood of the fitted model; LL₀ = Log-likelihood of the null model (intercept-only); McFadden’s R² = 1 − (LL/ LL₀), a pseudo-R² measure of goodness-of-fit; values closer to 1 indicate better fit.

### Sensitivity analysis

Similar effect values and consistent statistical significance were yielded in the sensitivity analysis of air pollution’s association with arthritis prevalence, except the association between PM_1_ exposure and arthritis prevalence in the population that had changed their residence (Tables 5–8). The OR value for PM_1_ remained at 1.069 when including participants who had changed their residence; however, the substantial increase in the p-value suggested that the association may exhibit greater variability or uncertainty in this group. Furthermore, we observed a notable improvement in the p-values for the association between air pollutants and arthritis prevalence when excluding the population who had changed their residence, with a corresponding enhancement in the OR values (Table 7). These results indicated that, even when considering sample heterogeneity or potential biases, the association between pollutants and arthritis remained significant, underscoring the reliability of the findings.

**Table 5:** Sensitivity Analysis of 2-Year Air Pollution Exposure and Arthritis.

Air pollution	2-year average			3-year average		
	IQR	OR and 95%CI	*P*	IQR	OR and 95%CI	*P*
NH_4_	0.131μg/m³	1.131(1.012,1.264)	0.029	0.5μg/m³	1.143(1.017,1.285)	0.024
NO_3_	0.209μg/m³	1.074(1.006,1.147)	0.032	0.87μg/m³	1.082(1.011,1.159)	0.022
PM_1_	0.575μg/m³	1.02(1.007,1.033)	0.009	2.51μg/m³	1.044(1.011,1.07)	0.005
PM_10_	2.35μg/m³	1.01(1.001,1.02)	0.022	8.46μg/m³	1.012(1.002,1.021)	0.014
PM_2.5_	1.204μg/m³	1.017(1.002,1.032)	0.021	4.70μg/m³	1.019(1.004,1.036)	0.013

Abbreviations: NH₄, Ammonium; NO₃, Nitrate; PM_1_, Particulate Matter 1; PM_10_, Particulate Matter 10; PM_2.5_, Particulate Matter 2.5; IQR, inter-quartile range; OR, odds ratio; 95% CI, 95% Confidence Interval.

**Table 6:** Sensitivity Analysis with Regional Adjustment: Air Pollution-Arthritis Association.

Air pollution	Regional variable was adjusted		Regional variable was not adjusted	
	OR and 95% CI	*P*-value	OR and 95% CI	*P*-value
NH_4_	1.145(0.997,1.314)	0.053	1.143(1.017,1.285)	0.024
NO_3_	1.084(0.999,1.176)	0.052	1.082(1.011,1.159)	0.022
PM_1_	1.042(1.009,1.077)	0.011	1.044(1.011,1.07)	0.005
PM_10_	1.011(1.001,1.021)	0.029	1.012(1.002,1.021)	0.014
PM_2.5_	1.02(1.002,1.038)	0.027	1.019(1.004,1.036)	0.013

Abbreviations: NH₄, Ammonium; NO₃, Nitrate; PM_1_, Particulate Matter 1; PM_10_, Particulate Matter 10; PM_2.5_, Particulate Matter 2.5; IQR, inter-quartile range; OR, odds ratio; 95% CI, 95% Confidence Interval.

**Table 7:** Sensitivity Analysis Excluding Residential Mobility: Pollution-Arthritis Link.

Air pollution (IQR)	Participants who had changed their address were included		Participants who had changed their address were excluded	
	OR and 95% CI	*P*-value	OR and 95% CI	*P*-value
NH_4_ (0.5 μg/m³)	1.143(1.017,1.285)	0.024	1.285(1.109,1.49)	< 0.001
NO_3_ (0.87 μg/m³)	1.082(1.011,1.159)	0.022	1.162(1.066,1.266)	< 0.001
PM_1_ (2.51 μg/m³)	1.044(1.011,1.07)	0.005	1.069(1.032,1.107)	1.647
PM_10_ (8.46 μg/m³)	1.012(1.002,1.021)	0.014	1.019(1.007,1.03)	< 0.001
PM_2.5_ (4.70 μg/m³)	1.019(1.004,1.036)	0.013	1.034(1.015,1.054)	< 0.001

Abbreviations: NH₄, Ammonium; NO₃, Nitrate; PM_1_, Particulate Matter 1; PM_10_, Particulate Matter 10; PM_2.5_, Particulate Matter 2.5; IQR, inter-quartile range; OR, odds ratio; 95% CI, 95% Confidence Interval.

**Table 8:** Sensitivity Analysis Using Log-Binomial Regression for Pollution-Arthritis Association.

Air pollution (IQR)	Logistic regression		Log-binomial Poisson regression	
	OR and 95% CI	*P*-value	OR and 95% CI	*P*-value
NH_4_ (0.5 μg/m³)	1.113(1.002,1.236)	0.044	1.143(1.017,1.285)	0.024
NO_3_ (0.87 μg/m³)	1.065(1.002,1.133)	0.042	1.082(1.011,1.159)	0.022
PM_1_ (2.51 μg/m³)	1.032(1.006,1.058)	0.013	1.044(1.011,1.07)	0.005
PM_10_ (8.46 μg/m³)	1.009(1.000,1.018)	0.029	1.012(1.002,1.021)	0.014
PM_2.5_ (4.70 μg/m³)	1.015(1.001,1.03)	0.027	1.019(1.004,1.036)	0.013

Abbreviations: NH₄, Ammonium; NO₃, Nitrate; PM_1_, Particulate Matter 1; PM_10_, Particulate Matter 10; PM_2.5_, Particulate Matter 2.5 Abbreviations: NH₄, Ammonium; NO₃, Nitrate; PM_1_, Particulate Matter 1; PM_10_, Particulate Matter 10; PM_2.5_, Particulate Matter 2.5; IQR, inter-quartile range; OR, odds ratio; 95% CI, 95% Confidence Interval.

## Discussion

This nationwide study on Chinese adults demonstrated a significant association between long-term PM_2.5_, PM_10_, and PM_1_ exposure and elevated arthritis prevalence. After controlling for confounding factors, NO_3_ and NH_4_ also showed a significant association with enhanced arthritis prevalence. Furthermore, NH_4_ and NO_3_ contributed the most to increasing arthritis prevalence, followed by PM_1_ and PM_2.5_, with PM_10_ being the least influential. However, the positive association between NH_4_ and NO_3_ became significant only in adjusted model 2, suggesting that their effects may depend on controlling for confounding factors. In contrast, the positive associations between PM_1_, PM_2.5_, and PM_10_ were significant across all models, highlighting the robustness and directness of their associations.

Ho et al. [29] observed that elevated PM₂.₅ concentrations significantly increase the risk of incident rheumatoid arthritis, and Chang et al. [30] identified NO₂ as an independent predictor of new‐onset RA. Although those studies focused on incidence and applied Cox proportional‐hazards models, our cross‐sectional analysis evaluated prevalence using generalized linear models; nevertheless, the direction and magnitude of the PM₂.₅ and NO_X_ associations are strikingly consistent across these different epidemiological designs, lending further support to a causal relationship between fine and gaseous pollutants and arthritis risk. Zhang et al. [17] additionally demonstrated in the UK Biobank that combined exposure to PM₁₀, PM₂.₅ and NO₂ interacts with elevated genetic susceptibility to markedly amplify incident RA risk—an observation that parallels our finding of particularly strong associations for NH₄ and NO₃ in fully adjusted models, and underscores the importance of secondary inorganic aerosols in future studies. Beyond PM₂.₅ and NO₂, ours is, to our knowledge, the first large‐scale study in a middle‐aged and elderly Chinese cohort to quantify the associations between PM₁, NH₄ and NO₃ and arthritis prevalence. Previous cardiovascular research linked NH₄ and NO₃ to increased hospitalization rates [15] and cardiopulmonary mortality [31], but comparable analyses in the arthritis literature are lacking. The odds ratios we observed for NH₄ and NO₃—particularly after comprehensive adjustment—suggest that these secondary inorganic aerosols may play a more important role in disease pathogenesis than previously appreciated. With respect to secondary and gaseous pollutants, we observed no significant association between O₃ and arthritis prevalence, aligning with Fitriyah et al. [32]. In contrast, our SO₄ findings diverged from He et al. [18], potentially due to: ① temporal variations in SO₄ precursor emissions; ② regional differences in land use affecting SO₄ exposure; ③ model specification disparities influencing effect estimates.

Although the mechanisms by which air pollution influences arthritis prevalence remain unclear, it may potentially promote the disease through three pathways: oxidative stress, immune imbalance, and gene–environment synergy. Inhaled PM₂.₅ and NO_X_ penetrate deeply into the alveoli [18], where overproduction of reactive oxygen species (ROS) and reactive nitrogen species (RNS) induces oxidative stress that injures airway and vascular endothelial cells and induces formation of inducible bronchus-associated lymphoid tissue (iBALT). Within the iBALT microenvironment, activated macrophages and dendritic cells engage the NF-κB pathway to secrete IL-1β, IL-6, and TNF-α [33], cytokines that enter the bloodstream and home to the synovium where they stimulate fibroblast-like synoviocyte proliferation and pathological angiogenesis, mechanisms that may manifest clinically as visible swelling of the metacarpophalangeal and proximal interphalangeal joints; these same cytokines upregulate matrix metalloproteinases and aggrecanases that degrade proteoglycan networks and collagen fibers, thereby leading to symmetric polyarticular pain and a progressive loss of range of motion; and through RANKL/RANK signaling they activate osteoclasts to resorb subchondral bone [34], which may contribute to joint-space narrowing and systemic features such as fatigue and low-grade fever. Secondly, exposure to air pollutants also notably affects immune system balance, triggering autoimmune responses. Particulate matter (such as PM₂.₅) induces bronchus-associated lymphoid tissue formation in the respiratory tract, activating antigen-presenting cells such as dendritic cells and enhancing T-cell and B-cell activation; this process facilitates generation of autoantibodies—anti-citrullinated protein antibodies (ACPA) and rheumatoid factor—whose elevated serum levels may indicate a higher risk of joint destruction, and disrupts immune tolerance, becoming a crucial early event in RA pathogenesis [23]. Additionally, pollutants exacerbate the inflammatory response by enhancing T-helper 17 cell viability while suppressing regulatory T-cell function [21], leading to immune complex–mediated synovitis that may manifest as persistent swelling and tenderness of small joints (e.g., metacarpophalangeal and proximal interphalangeal joints); Th17-driven osteoclast activation via the RANKL/RANK pathway and pannus invasion may result in ulnar deviation and swan-neck deformities; and systemic inflammation may give rise to extra-articular features such as rheumatoid nodules, interstitial lung disease, or vasculitis, reflecting the systemic nature of the autoimmune response. Finally, gene–environment interplay amplifies these effects. In HLA-DRB1 “shared epitope” carriers, PM₂.₅/NO_X_ exposure enhances PAD-driven citrullination in the lung, and shared epitopes preferentially present citrullinated peptides to CD4 ⁺ T cells, breaking tolerance and accelerating ACPA production [35–37].

Based on the above findings, we propose a multi-stage lung-to-joint pathogenic axis: chronic inhalation of PM₂.₅ and NO_X_ induces oxidative stress and iBALT formation in the alveoli, where activated macrophages and dendritic cells release TNF-α, IL-1β and IL-6 via NF-κB. These cytokines then enter the bloodstream and preferentially localize to the synovium, driving synoviocyte proliferation, angiogenesis and extracellular matrix degradation. Concurrently, PAH-mediated activation of the aryl hydrocarbon receptor in the lung skews dendritic cells toward a Th17 phenotype while suppressing regulatory T cells, thereby promoting B-cell affinity maturation and the generation of ACPA, which deposit in the joint and amplify local inflammation. In individuals carrying HLA-DRB1 shared-epitope alleles, pollutant-induced PAD activity in the pulmonary mucosa enhances protein citrullination and the presentation of citrullinated peptides to CD4⁺ T cells, breaking peripheral tolerance and accelerating ACPA production. Together, these sequential pulmonary and systemic events converge to initiate and drive the development of arthritis.

Our study offers several notable advantages. Firstly, this article investigated the association of various environmental air pollutants (predominantly particulate matter) with arthritis prevalence. Unlike previous studies that primarily focused on PM_2.5_, we also explored the impacts of PM_1_, PM_10_, NH_4_, and NO_3_, providing a more comprehensive understanding of the connection between particulate pollution and arthritis. Furthermore, our study did not rely on fixed environmental monitoring stations; instead, we utilized satellite remote sensing, as well as innovative statistical methods to estimate pollutant concentrations with a spatial resolution of approximately 10 kilometers. This approach may offer advantages in terms of accuracy and resolution. Finally, sensitivity analyses were conducted, demonstrating the robustness of the findings. However, certain limitations exist in this research. First, McFadden’s pseudo R² values ranged from 0.02 to 1, suggesting that the model fit could still be improved. Second, meteorological factors (such as temperature and humidity) were not accounted for, potentially influencing the association between air pollution and arthritis prevalence. More related confounders should be controlled in future research to validate our results. However, certain limitations exist in this research. First, McFadden’s pseudo R² values ranged from 0.02 to 1, suggesting that the model fit could still be improved, and our extensive dichotomization of covariates may have obscured the original effect gradients. Second, meteorological factors (such as temperature and humidity) were not accounted for, potentially influencing the association between air pollution and arthritis prevalence. Third, our exposure assessment based on CHAP’s fixed‐site gridded interpolation provides dense coverage in urban and county centers but lower spatial resolution in remote and rural areas, cannot capture individual‐level variation due to indoor environments, commuting routes, or temporary relocations, and multi‐year daily averages may smooth out short‐term pollution peaks, thereby underestimating acute exposure effects. Fourth, because exposure levels and disease status were assessed simultaneously, our cross‐sectional design may be subject to reverse‐causation bias. Fifth, prevalence estimates are vulnerable to survivor bias, as individuals with higher exposure levels or more severe disease may die or be lost to follow‐up before the survey, enriching the sample for long‐term survivors with milder disease or lower exposures and attenuating the true exposure–disease association. Finally, CHARLS defines arthritis by a single self‐reported question without distinguishing OA from RA, limiting our ability to attribute pollutant–arthritis associations to specific subtypes. Given that the global prevalence of OA is approximately 3.8% compared with 0.24% for RA, most of the 520 self-reported cases in our sample likely represent OA. Epidemiological research has predominantly focused on RA, leaving human data on OA scarce; this limitation prevents assessment of whether long‐term particulate exposure affects OA and RA differently. Future studies should distinguish between OA and RA populations to clarify their respective responses to ambient particulate matter.

## Conclusion

In middle-aged and elderly Chinese adults, long-term exposure to fine and coarse particulate matter and secondary inorganic aerosols was significantly associated with higher self-reported arthritis prevalence, albeit with modest odds ratios. Given the cross-sectional design, reliance on self-reported outcomes, potential survivor and reverse-causation biases, and limited adjustment for meteorological and individual-level confounders, these associations warrant cautious interpretation. Future longitudinal studies with precise exposure assessment and objective clinical endpoints are required to validate and clarify these findings.

### Consent for publication

The author confirms that the contents of this manuscript have not been copyrighted or published previously, are not currently under consideration for publication elsewhere, and will not be copyrighted, submitted, or published elsewhere while under consideration by PLOS one.
